# Neuromyelitis optica spectrum disorders with and without connective tissue disorders

**DOI:** 10.1186/s12883-018-1182-5

**Published:** 2018-10-24

**Authors:** Chun-Sheng Yang, Qiu Xia Zhang, Sheng Hui Chang, Lin Jie Zhang, Li Min Li, Yuan Qi, Jing Wang, Zhi Hua Sun, Nannan Zhangning, Li Yang, Fu-Dong Shi

**Affiliations:** 1 Department of Neurology, Tianjin Neurological Institute, Tianjin Medical University General Hospital, No 154 Anshan Road, Heping District, Tianjin, 300052 China; 2 Department of Radiology, Tianjin Medical University General Hospital, No 154 Anshan Road, Heping District, Tianjin, 300052 China; 3 Department of Neurology, Barrow Neurological Institute, St. Joseph’s Hospital and Medical Center, Phoenix, AZ 85013 USA

**Keywords:** Neuromyelitis optica, Neuromyelitis optica spectrum disorders, Connective tissue disorders, Autoantibodies, Magnetic resonance imaging

## Abstract

**Background:**

Neuromyelitis optica spectrum disorders (NMOSD) often coexist with connective tissue disorders (CTD). The aim of this study was to investigate and compare the features of NMOSD with and without CTD.

**Methods:**

NMOSD patients with (*n* = 18) and without CTD (*n* = 39) were enrolled, and the clinical, laboratory, and magnetic resonance imaging (MRI) features of the two groups were assessed.

**Results:**

Most of the demographic and clinical features examined were similar between NMOSD patients with and without CTD. Serum immunoglobulin G (IgG), percentage of γ-globulin and seropositivity for several other autoantibodies were significantly elevated in NMOSD patients with CTD (*P* < 0.05). NMOSD with CTD was marked by longer spinal cord lesions and a lower frequency of short transverse myelitis (TM) than NMOSD without CTD (*P* < 0.05). NMOSD with CTD also featured more T1 hypointensity and T2 bright spotty lesions (BSLs) on MRI than NMOSD without CTD (*P* = 0.001 and 0.011, respectively). There were no other differences in laboratory, MRI and clinical characteristics between different NMOSD subtypes.

**Conclusions:**

A few characteristics differed between NMOSD with and without CTD. NMOSD patients with CTD had higher serum IgG, longer spinal cord lesions, a lower frequency of short TM and more T1 hypointensity and T2 BSLs on spinal MRI than NMOSD patients without CTD.

## Background

Neuromyelitis optica spectrum disorders (NMOSD) is a severe central nervous system (CNS) demyelinating syndrome, characterized by optic neuritis (ON) and acute myelitis [1]. Immunoglobulin G autoantibodies against aquaporin-4 (AQP4-IgG) play a key role in the pathogenesis and diagnosis of NMOSD. Standardized diagnostic criteria for NMOSD were published in 2015, further stratifying NMOSD based on serologic testing (NMOSD with or without AQP4-IgG). According to these criteria, NMOSD has six core clinical characteristics: ON, acute myelitis, area postrema syndrome, acute brainstem syndrome, acute diencephalic clinical syndrome and symptomatic cerebral syndrome [2].

In addition to AQP4-IgG, antinuclear autoantibodies (ANAs) are also often detectable in patients with NMOSD who do not have clinical evidence of a systemic autoimmune disease [3]. However, an increasing number of reports have revealed that NMOSD often coexists with connective tissue disorders (CTD), particularly systemic lupus erythematosus (SLE) and Sjögren syndrome (SS) [3–5]. Whether there are clinically significant differences in NMOSD with and without CTD remains unclear. In this study, we investigated and compared the demographic, clinical, laboratory, and magnetic resonance imaging (MRI) characteristics of NMOSD with and without CTD.

## Methods

### Patients

Through the hospital database, we reviewed the records of all NMOSD patients admitted to Tianjin Medical University General Hospital, Tianjin, China, from December 2014 to December 2017. The mean follow-up time was 5.27 (0.42–19) years. NMOSD was diagnosed according to the 2015 international consensus diagnostic criteria for NMOSD [2], and CTD was diagnosed by rheumatologists according to published criteria and typology guidelines (e.g., SLE [6], SS [7], rheumatoid arthritis (RA) [8], or undifferentiated CTD (UCTD) [9]). Other inclusion criteria were as follows: (a) the serum samples of all the patients were tested for AQP4-IgG, myelin oligodendrocyte glycoprotein immunoglobulin G (MOG-IgG), autoreactive antibodies ((ANAs), extractable nuclear antigen autoantibodies (ENAs), rheumatoid factor (RF), and anti-neutrophil cytoplasmic antibodies (ANCAs)), immunoglobulins, and complement (C); (b) spinal and brain MRI were available before high-dose intravenous methylprednisolone (IVMP) (1.0 g/d for 3 days) or intravenous immunoglobulin G (IVIG) (0.4 g/kg.d for 5 days); and (c) the time between study inclusion and the last relapse was more than 3 months. We excluded MOG-IgG positive patients, since the pathophysiology of MOG-IgG associated NMOSD is probably different in comparison to AQP4-IgG positive NMOSD. The information of personal accounts and clinical signs, Kurtzke Expanded Disability Status Scale (EDSS) scores, blood and cerebrospinal fluid (CSF) laboratory data, and MRI were recorded in our databank. The EDSS was applied before high-dose IVMP or IVIG during relapse and during the remission period by two neurologists, both certified by Neurostatus for EDSS competency. The database comprised 74 Chinese patients diagnosed with NMOSD during the period. A total of 17 patients were excluded: 10 didn’t have adequate data available, and another 7 were MOG-IgG positive. Ultimately, 39 NMOSD patients without CTD and 18 NMOSD patients with CTD were recruited in the cohort.

The study was approved by the Ethics Committee of Tianjin Medical University General Hospital, and written informed consent was obtained from each participant.

### Laboratory testing

AQP4-IgG tests, MOG-IgG tests and CSF oligoclonal banding (OCB) were conducted in our clinical neuroimmunological laboratory. AQP4-IgG was detected by a cell-based assay (CBA), which has been described previously [10]. The plasmids were donated by Professor Angela Vincent and Professor David Beeson, Nuffield Department of Clinical Neurosciences, University of Oxford. Tests for autoreactive antibodies (ANAs, ENAs, RF and ANCAs), immunoglobulins, and complement were conducted, along with other serological profiling, in the immunology and clinical laboratory of our hospital.

### MRI

MRI was performed using either a 1.5 T or a 3 T magnet from one of two manufacturers: GE (GE Medical Systems, Milwaukee, WI, USA) or Siemens (Siemens AG, Erlangen, Germany). Routine spinal MRI included T1-weighted imaging (T1WI), T2-weighted imaging (T2WI), a sagittal short tau inversion recovery (STIR) sequence, and T1WI with gadolinium enhancement. The T1 signal intensity of the lesion and the appearance of bright spotty lesions (BSLs) on T2 were recorded. ‘T1 dark’ was defined when the signal intensity of the lesion was similar to that of CSF on the T1WI. BSLs were defined when the signal intensity of the lesion approached that of the surrounding CSF without flow-void effects on the T2WI, as previously described [11, 12]. On axial T2WI, lesion distribution was classified as: ‘peripherally- located’ or ‘centrally- located’. Lesions that were ≥ 50% of the spinal cord area (transverse myelitis, TM) were noted. Short TM (< 3 vertebral segments) was also recorded. The brain MRI protocol included diffusion-weighted images, T1WI, T2WI, fluid-attenuated inversion recovery (FLAIR) imaging, and contrast-enhanced T1WI. The slice thickness of the axial scans was 5 mm. MRI lesions were evaluated independently by two radiologists blinded to the patients’ information.

### Statistical analysis

Statistical analysis was performed using the Statistical Package for the Social Sciences (SPSS 22.0). All quantitative data in this study were presented as the mean ± standard deviation (SD) or the median and range. We applied the Mann–Whitney U test for quantitative data and the chi-squared test or Fisher’s exact test for qualitative data. The relationships between variables were analysed using Spearman’s correlation coefficient and partial correlation analysis. *P*-values of < 0.05 were considered statistically significant.

## Results

From 2014 to 2017, a total of 57 patients satisfied the diagnostic criteria for inclusion in this study: 39 NMOSD patients without CTD and 18 with CTD (including 7 with SS, 3 with SLE, 1 with RA, and 7 UCTD). The demographic and clinical features of NMOSD with CTD are summarized in Table 1. Among the 18 patients with CTD, 9 developed CTD before NMOSD (ranging from 2 months to 30 years), and in the other 9 patients, the diagnosis of CTD followed the diagnosis of NMOSD (ranging from 1 month to 5 years). The demographic and clinical features of the patients are summarized in Table 2. Regular therapy was defined as a full dose of immunosuppressants. NMOSD patients with CTD had higher EDSS scores and more severe sensory disability at nadir than NMOSD patients without CTD (*P* < 0.05). No other significant demographic or clinical features difference was found in between NMOSD patients with and without CTD (*P* >  0.05).

**Table 1:** Demographic and clinical characteristics of NMOSD with CTD

	SS(7)	UCTD (7)	SLE(3)	RA(1)
Gender, *n* (% female)	7 (100%)	7 (100%)	3 (100%)	1 (100%)
Age at onset, years	41.29 ± 11.73	39.29 ± 9.07	40.67 ± 11.15	45.0 ± 0
Follow-up duration, years	6.07 ± 4.44	5.54 ± 3.79	2.51 ± 1.96	2.0 ± 0
Annualized relapse rate (ARR)	1.87 ± 2.36	0.92 ± 0.51	1.40 ± 0.45	1.5 ± 0
neuropathic pain, *n* (%)	3 (30%)	6 (60%)	1 (10%)	0 (0%)
Number of attacks	5.29 ± 2.69	3.71 ± 1.70	3.67 ± 3.06	3.0 ± 0
EDSS at nadir	5.29 ± 2.84	4.21 ± 1.82	6.17 ± 2.36	3.0 ± 0
EDSS at last follow-up	4.07 ± 2.99	2.21 ± 0.57	4.17 ± 2.47	2.0 ± 0
Initial presentation, *n* (%)				
ON	2 (28.57%)	2 (28.57%)	1 (33.33%)	1 (100%)
Area postrema syndrome	2 (28.57%)	0 (0%)	0 (0%)	0 (0%)
AM	2 (28.57%)	5 (71.43%)	2 (66.67%)	0 (0%)
Others	1 (14.29%)	0 (0%)	0 (0%)	0 (0%)

*Abbreviations: NMOSD* neuromyelitis optica spectrum disorders, *CTD* connective tissue disorders, *SS* Sjögren syndrome, *UCTD* undifferentiated connective tissue disorders, *SLE* systemic lupus erythematosus, *RA* rheumatoid arthritis, *EDSS* Kurtzke Expanded Disability Status Scale, *ON* optica neuritis, *AM* acute myelitis

**Table 2:** Demographic and clinical characteristics of NMOSD with and without CTD

	NMOSD (39)	NMOSD with CTD (18)	*P*
Gender, *n* (% female)	37 (94.9%)	18 (100%)	0.839
Age at onset, years	39.97 ± 13.82	42.33 ± 11.29	0.530
Follow-up duration, years	5.63 ± 4.63	5.04 ± 3.83	0.641
Annualized relapse rate (ARR)	0.98 ± 0.54	1.40 ± 1.51	0.127
ARR before regular medication	1.58 ± 2.18	1.66 ± 1.44	0.899
ARR after regular medication	0.64 ± 0.79	1.39 ± 1.96	0.100
Number of attacks	3.97 ± 2.07	4.28 ± 2.32	0.623
Neuropathic pain, *n* (%)	21 (53.8%)	10 (55.6%)	0.904
EDSS at nadir	3.5 (1, 8)	4 (1.5, 8.5)	0.031*
Visual functions	0 (0, 6)	1 (0, 6)	0.139
Pyramidal functions	1 (0, 4)	2 (0, 4)	0.219
Sensory functions	2 (0, 4)	3 (0, 4)	0.007*
Bowel and bladder	0 (0, 5)	3 (0, 5)	0.256
EDSS at last follow-up	2 (1, 8)	2.5 (1.5, 8.5)	0.403
Visual functions	0 (0, 4)	1 (0, 6)	0.111
Pyramidal functions	1 (0, 4)	1 (0, 4)	0.595
Sensory functions	1 (0, 4)	2 (0, 4)	0.063
Bowel and bladder	0 (0, 5)	0 (0, 5)	0.856
Initial presentation, *n* (%)			
ON	14 (35.9%)	6 (33.3%)	0.850
Area postrema syndrome	8 (20.5%)	2 (11.1%)	0.622
AM	15 (38.5%)	9 (50.0%)	0.412
Others	2 (5.1%)	1 (5.6%)	1.000

*Abbreviations: NMOSD* neuromyelitis optica spectrum disorders, *CTD* connective tissue disorders, *EDSS* Kurtzke Expanded Disability Status Scale, *ON* optica neuritis, *AM* acute myelitis

*P < 0.05

The laboratory features of the patients are summarized in Table 3. CSF white blood cell (WBC) counts, protein, and chloride (Cl) showed no significant difference between the two groups. The level of serum IgG was significantly higher in NMOSD patients with CTD than those without CTD (*P* = 0.013), and a similar result was found for the percentage of γ-globulin (*P* = 0.023). Furthermore, ANA, anti-SSA/Ro antibodies (anti-SSA), anti-SSB/La antibodies (anti-SSB), anti-Ro52, and RF were significantly higher in NMOSD patients with CTD than in those without CTD (*P* < 0.05). However, there was no difference between the two groups in the positivity rate of AQP4-IgG, OCB, CRP (C-reactive protein), IgE, anti-dsDNA (anti-double stranded DNA antibodies), anti-nRNP (antinuclear ribonucleoprotein), anti-Sm (anti-Sm antibodies), anti-Scl70 (anti-topoisomerase I antibodies), anti-Jo1 (anti-Jo-1 antibodies), ACA (anti-neutrophil cytoplasmic antibodies), AnuA (anti-nucleosome antibody), AHA (anti-histone antibody), ARPA (anti-ribonucleoprotein antibodies), or GPI (Glucose-6 phosphate isomerase). No significant difference was found in the level of IgA, IgM, C3, or C4 between the two groups of patients.

**Table 3:** Laboratory features between NMOSD with and without CTD

	NMOSD (39)	NMOSD with CTD (18)	*P*
CSF Index			
Elevated white cell count (> 8 × 10^6^/L), *n* (%)	5 (12.8%)	5 (27.8%)	0.315
Elevated protein (> 0.4 g/L), *n* (%)	12 (30.8%)	5 (27.8%)	0.819
OCB, *n* (%)	1 (2.6%)	1 (5.6%)	1.000
Glu (2.5–4.4 mmol/L)	3.69 ± 1.00	3.19 ± 1.30	0.137
Cl (119-130 mmol/L)	127.25 ± 5.32	126.29 ± 5.41	0.585
Serums Index			
AQP4-Ab, *n* (%)	29 (74.4%)	12 (66.7%)	0.548
IgG (751–1560 mg/dl)	1161.73 ± 393.18	1696.06 ± 760.54	0.013*
IgA (82-453 mg/dl)	220.90 ± 95.81	372.66 ± 290.69	0.051
IgM (46–304 mg/dl)	114.47 ± 64.61	89.41 ± 35.85	0.144
C3 (79–152 mg/dl)	104.64 ± 67.35	99.29 ± 17.12	0.750
C4 (16–38 mg/dl)	23.20 ± 16.12	22.24 ± 9.92	0.822
CRP (> 0.8 mg/dl), *n* (%)	4 (10.3%)	6 (33.3%)	0.079
IgE (> 165 IU/ml), *n* (%)	2 (5.1%)	1 (5.6%)	1.000
ANA (> 1:80), *n* (%)	22 (56.4%)	17 (94.4%)	0.004*
Anti-dsDNA, *n* (%)	1 (2.6%)	1 (5.6%)	1.000
Anti-nRNP, *n* (%)	0 (0.0%)	1 (5.6%)	–
Anti-Sm, *n* (%)	0 (0.0%)	1 (5.6%)	–
Anti-SSA, *n* (%)	10 (25.6%)	15 (83.3%)	< 0.001**
Anti-Ro52, *n* (%)	9 (23.1%)	13 (72.2%)	< 0.001**
Anti-SSB, *n* (%)	2 (5.1%)	8 (44.4%)	0.001*
Anti-Scl70, *n* (%)	0 (0.0%)	0 (0.0%)	–
Anti-Jo1, *n* (%)	0 (0.0%)	0 (0.0%)	–
ACA, *n* (%)	0 (0.0%)	0 (0.0%)	–
AnuA, *n* (%)	0 (0.0%)	3 (16.7%)	–
AHA, *n* (%)	2 (5.1%)	2 (11.1%)	0.792
ARPA, *n* (%)	0 (0.0%)	0 (0.0%)	–
GPI (> 0.20 mg/L), *n* (%)	2 (5.1%)	1 (5.6%)	1.000
RF (> 20 IU/ml), *n* (%)	3 (7.7%)	7 (38.9%)	0.012*
ASO (> 116 IU/ml), *n* (%)	4 (10.3%)	3 (16.7%)	0.802
globulin (53.8–68.2)	66.91 ± 3.43	63.07 ± 6.61	0.032*
α1 globulin (1.1–3.7%)	2.17 ± 0.52	2.46 ± 1.45	0.328
α2 globulin (8.5–14.5%)	9.05 ± 1.35	8.97 ± 1.40	0.843
βglobulin (8.6–14.8%)	8.92 ± 1.51	8.97 ± 2.22	0.926
γglobulin (9.2–18.2%)	12.95 ± 3.13	16.87 ± 6.35	0.023*

*Abbreviations: NMOSD* neuromyelitis optica spectrum disorders, *CTD* connective tissue disorders, *CSF* cereberal spinal fluid, *OCB* oligoclonal bands, *Glu* glucose, *Cl* chloride, *C* complements, *CRP* C-reactive protein, *ANA* antinuclear antibodies, *Anti-dsDNA* anti-double stranded DNA antibodies, *Anti-nRNP* antinuclear ribonucleoprotein, *Anti-Sm* anti-Sm antibodies, *Anti-SSA/Ro52/SSB* Anti-SSA/Ro52/SSB antibodies, *Anti-Scl70* anti-topoisomerase I antibodies, *Anti-Jo1* anti-Jo-1 antibodies, *ACA* anti-neutrophil cytoplasmic antibodies, *AnuA* anti-nucleosome antibody, *AHA* anti-histone antibody, *ARPA* anti-ribonucleoprotein antibodies, *GPI* Glucose-6 phosphate isomerase, *RF* rheumatoid factor, *ASO* Anti-streptolysin

**P < 0.001, *P < 0.05

**Fig. 1:**
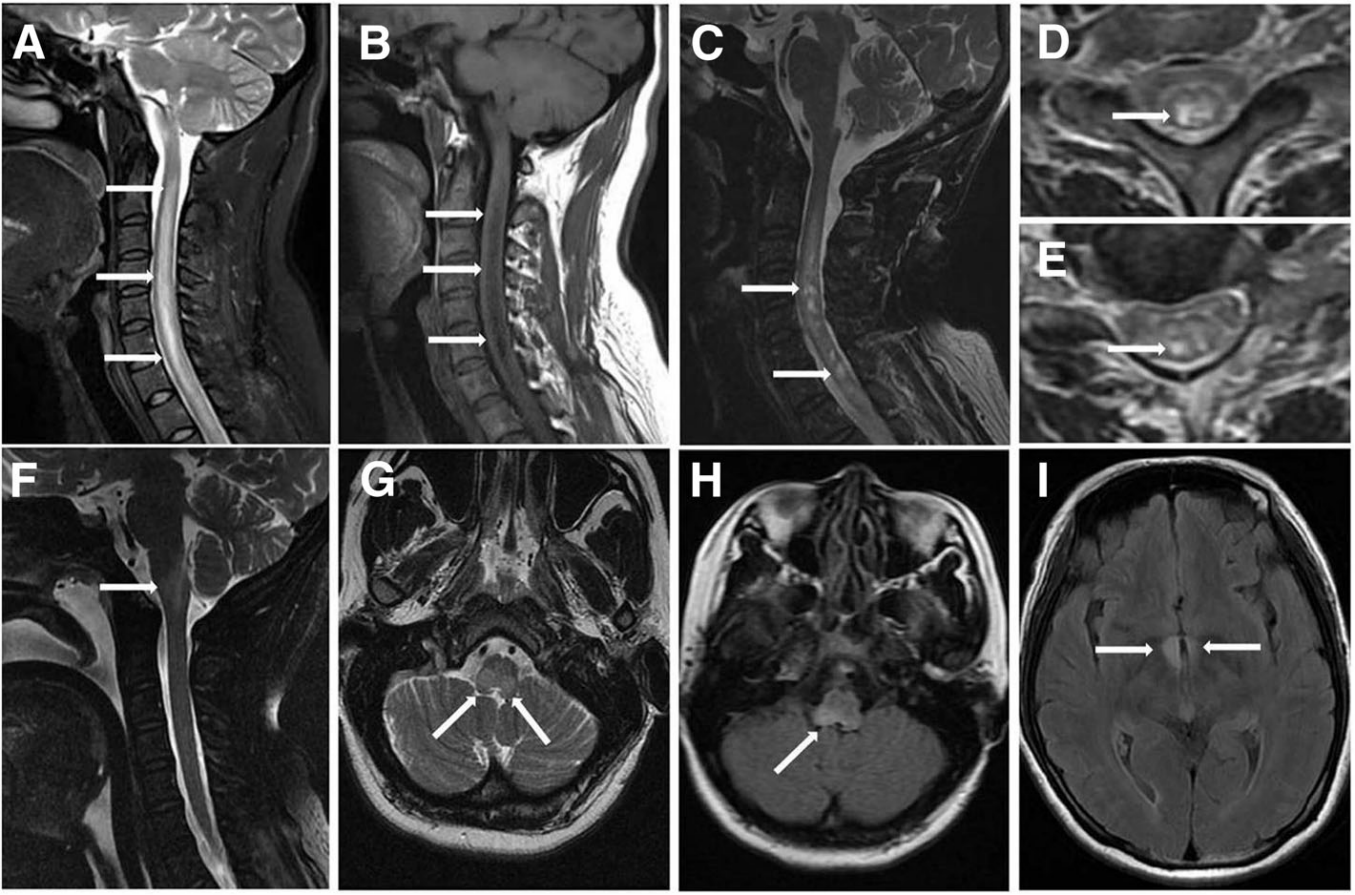
Representative MRI abnormalities (arrows) in patients with NMOSD with CTD. **a** and **b** are from a 35-year-old woman with NMOSD and SS; (**a**) shows longitudinally extensive transverse myelitis (LETM) lesions on T2WI, and **b** shows ‘T1 dark’ associated with LETM. **c**, **d** and **e**, from a 40-year-old woman with NMOSD and SLE, show bright spotty lesions (BSLs) associated with LETM on T2WI. **f**, from a 38-year-old woman with NMOSD and SS, shows an area postrema lesion on T2WI. **g**, A 45-year-old woman with RA, shows a medulla oblongata lesion on T2WI. **h**, from a 39-year-old woman with NMOSD and undifferentiated CTD (UCTD), shows an area postrema lesion on FLAIR imaging. **i**, A 45-year-old woman with NMOSD and UCTD, showed bilateral hypothalamus lesions on the FLAIR imaging

The spinal and brain MRI features of the patients are summarized in Tables 4 and 5, respectively. Representative MRI abnormalities (arrows) in NMOSD patients with CTD are shown in Fig. 1. The length of spinal cord lesions was longer in NMOSD patients with CTD than in NMOSD patients without CTD (*P* = 0.018). There was no significant difference in the frequency of short TM at onset between the two groups. However, the frequency of short TM at the initial manifestation of myelitis was significantly higher in NMOSD patients without CTD than in those with CTD (*P* = 0.010). The frequency of T1 hypointensity and T2 BSLs in acute myelitis was higher in NMOSD patients with CTD than in those without CTD (*P* = 0.001 and 0.011, respectively). No significant difference between the two groups was observed in any of the other MRI features.

**Table 4:** Spinal MRI features between NMOSD with and without CTD

	NMOSD (39)	NMOSD with CTD (18)	*P*
Sagittal location			
length of lesions (VB)	4.44 ± 2.89	7.56 ± 4.79	0.018*
Short TM at onset, *n* (%)	7 (17.9%)	1 (5.6%)	0.400
Initial short TM, *n* (%)	18 (46.2%)	2 (11.1%)	0.010*
Location of spinal lesions, *n* (%)			
Cervical cord	13 (33.3%)	2 (11.1%)	0.148
Cervico-thoracic cord	16 (41.0%)	10 (55.6%)	0.306
Thoracic cord	10 (25.6%)	6 (33.3%)	0.548
Axial location, *n* (%)			
Centrally located	38 (97.4%)	18 (100.0%)	1.000
Peripherally located	1 (2.6%)	0 (0.0%)	1.000
enhancement	11 (28.2%)	8 (44.4%)	0.227
Acute phase			
T1 dark, *n* (%)	17 (43.6%)	16 (88.9%)	0.001*
T2 BSLs, *n* (%)	14 (35.9%)	13 (72.2%)	0.011*
Chronic phase			
Fragmentation, *n* (%) or ‘bead-like’ lesions	25 (64.1%)	8 (44.4%)	0.162
Disappearance, *n* (%)	9 (23.1%)	7 (38.9%)	0.217
Atrophy, *n* (%)	5 (12.8%)	3 (16.7%)	1.000

*Abbreviations: NMOSD* neuromyelitis optica spectrum disorders, *CTD* connective tissue disorders, *VB* vertebral segments, *TM* transverse myelitis, *BSLs* bright spotty lesion

*P < 0.05

**Table 5:** Brain MRI features at onset between NMOSD with and without CTD

	NMOSD (39)	NMOSD with CTD (18)	*P*
Brain lesions, *n* (%)	14 (35.9%)	9 (50.0%)	0.313
Brain lobes	5 (12.8%)	5 (27.8%)	0.315
Basal ganglia	0 (0)	3 (16.7%)	–
Hypothalamic and thalamic	1 (2.6%)	0 (0)	–
Callosum	0 (0)	1 (5.6%)	–
Midbrain	1 (2.6%)	1 (5.6%)	1.000
Pons	1 (2.6%)	0 (0)	–
Medulla oblongata	8 (20.5%)	2 (11.1%)	0.622
Area postrema	8 (20.5%)	2 (11.1%)	0.622

*Abbreviations: NMOSD* neuromyelitis optica spectrum disorders, *CTD* connective tissue disorders

Pearson correlation results showed that EDSS scores were positively correlated with group classification (NMOSD with or without CTD) (*r* = 0.286, *P* = 0.031), the length of spinal cord lesions (*r* = 0.488, *P* < 0.001) and T1 hypointensity (*r* = 0.362, *P* = 0.006). EDSS scores showed no correlation with T2 BSLs (*r* = 0.172, *P* = 0.202) or AQP4-IgG positivity status (*r* = − 0.117, *P* = 0.388). However, partial correlation results showed that EDSS scores had no correlation with group classification after controlling for lesion length and T1 hypointensity (*r* = 0.003, *P* = 0.985).

## Discussion

In the present study, we found that patients with NMOSD and CTD were similar to those without CTD in all tested demographic and clinical features except EDSS scores, especially sensory disability at nadir. Furthermore, most clinical, laboratory, and MRI features also did not show significant differences between the two groups. However, a number of autoantibodies, CSF indexes, and MRI features differed significantly.

NMOSD patients with CTD had increased amounts of T1 hypointensity and T2 BSLs on spinal MRI in acute myelitis. T1 hypointensity and T2 BSLs probably indicated intense damage of the spinal cord [11, 12]. However, the characteristics of spinal MRI did not show any significant difference between the two groups in the chronic phase. These findings may partially explain the differences in sensory disability and EDSS scores at nadir. Since CTDs can cause peripheral neuropathy leading to sensory deficits, electromyography should be performed to exclude that diagnosis. None of the patients in the present study showed clinical symptoms or signs caused by peripheral neuropathy until the last follow-up, according to their records. Pain is common in NMOSD and can lead to a reduced quality of life [13, 14]. In the present study, neuropathic pain (NP) was defined carefully according to previous reports [13, 15]. Almost half of the patients in both groups complained of NP in the present study, the characteristics and intensity of this pain should be evaluated in the future using previously reported methods [13].

The serum autoantibody AQP4-IgG is highly sensitive and specific for NMOSD [16]. In 2004, Lennon and colleagues reported this autoantibody as a frequent feature of NMOSD; the presence of the antibody was 73% sensitive and 91% specific for clinically defined neuromyelitis optica (NMO) [17]. Since then, many study groups have tested AQP4-IgG in patients with NMO or NMOSD, with the reported frequency varying according to the assay, e.g., immunofluorescence, immunoprecipitation, radioimmunoprecipitation or CBA [18–22]. The new diagnostic criteria for NMOSD recommended CBA for the detection of AQP4-IgG because of its good sensitivity and specificity. Hence, we adopted CBA to detect AQP4-IgG in the present study. The seroprevalence of AQP4-IgG was 74.4% and 66.7% in patients with and without CTD, respectively, with no significant difference. This result was similar to those of Jarius et al. [23], who found that AQP4-IgG seropositivity in patients with CTD and co-existing neurological disorders was restricted to those with NMOSD [23]. This finding strongly suggests that AQP4-IgG was linked to the pathogenesis of NMOSD in those patients.

Although the frequency of ANAs was significantly higher in NMOSD patients with CTD than in those without CTD in our study, the frequency of ANAs in the latter group was more than 50%, which was similar to the frequencies reported in other reports [5, 11, 23–26]. Until now, the exact relationship between NMOSD and CTD has been unknown. A prevailing notion of NMOSD pathogenesis is that it is a complication of CTD manifested predominantly in the CNS. If a complication of CTD were the cause of NMOSD, one would expect that CTD would consistently be diagnosed before the onset of NMOSD. However, of the 18 NMOSD patients with CTD in our study, only 9 developed CTD before NMOSD, and in the other 9, the diagnosis of CTD followed the diagnosis of NMOSD, which was similar to previously reported data [5]. This chronology may support the current hypothesis that NMOSD and CTD can be co-existing conditions that are clinically expressed in patients susceptible to autoimmunity [27]. In the present study, the level of serum IgG and the percentage of γ-globulin were significantly higher in patients with CTD than in those without CTD, which was in agreement with a previous study [28]. Zhang and co-workers also found higher levels of CRP in NMOSD patients with autoimmune diseases than in those without autoimmune diseases. These findings may suggest that NMOSD patients with CTD have an intensified autoimmune response.

Longitudinally extensive transverse myelitis (LETM) lesions on spinal cord MRI are regarded as one of the six core clinical characteristics of NMOSD. The spinal cord lesions of NMOSD patients with CTD were longer than those of NMOSD patients without CTD. An increased frequency of both ‘T1 dark’ and T2 BSLs was found in NMOSD patients with CTD compared to those without CTD, in contrast to the findings of another study [28]. As previously reported, ‘T1 dark’ signals on spinal MRI was relatively specific to NMOSD, and were rarely found in MS [11, 12]. This feature may indicates early necrosis and cavity formation caused by intense cord damage. In previous reports, BSLs on axial T2WI were the most distinctive finding of NMO on spinal MRI, with frequencies of 54% and 86.1%, respectively [11, 12]. The pathophysiology of BSLs remains unclear. Compared to BSL-negative patients, BSL-positive patients had a higher frequency of contrast-enhanced lesions. Hence BSLs probably reflect severe damage to the spinal cord, which may destroy the blood-brain barrier (BBB) [11]. These significant differences between the two groups may also indicate that NMOSD patients with CTD are prone to intense autoimmune responses, which is in accordance with the elevated IgG, increased EDSS scores at nadir and intensified sensory disability in these patients compared to those without CTD. CTD-induced tissue damage probably promotes AQP4-IgG-induced pathology. ANAs or other inflammatory mechanisms in CTD may contribute to vascular damage and disruption of the BBB induced by vasculitis, which makes the AQP4-IgG accessible to the CNS and triggers the AQP4-IgG-mediated inflammatory cascade [23, 27]. EDSS scores were positively correlated with group classification (NMOSD and NMOSD with CTD), the length of spinal cord lesions and T1 hypointensity. However, partial correlation results showed that EDSS had no correlation with different groups after adjusting for the length of spinal cord lesions and T1 hypointensity. Therefore, the elevated EDSS scores in NMOSD patients with CTD may be due to the increased length of spinal cord lesions and frequency of T1 hypointensity. The results may also be influenced by other factors, which we will explore and investigate in the future.

Short TM (< 3 vertebral segments) is considered non-characteristic of NMOSD. In our study, we found that short TM is not uncommon in NMOSD. A short TM episode as an onset symptom was found in 17.9% of NMOSD patients without CTD and 5.6% of those with CTD, which were higher than those published in a previous report [29]. This difference was probably because Flanagan’s study included only AQP4-IgG-positive NMOSD cases. The frequency of short TM at the initial manifestation of myelitis was lower in NMOSD patients with CTD than in those without CTD, which may also indicate that NMOSD patients with CTD have a more intense autoimmune response. This finding is also consistent with the elevated levels of IgG and EDSS at nadir in NMOSD patients with CTD. Short TM can be the first presentation of NMOSD, which may delay diagnosis and treatment. Therefore, short TM, especially complicated with ‘T1 dark’ and/or T2 BSLs, should not exclude consideration of AQP4-IgG and ANAs testing, which may be helpful for avoiding delayed diagnosis and treatment of NMOSD.

Brain MRI in NMOSD is historically thought to be normal or non-specific, especially at the onset of the disease. However, the presence of certain lesions described as specific to NMOSD may be helpful in its diagnosis. Classic NMOSD lesions are those located at sites of high AQP4 expression, such as the periependymal areas, which include the hypothalamus, periaqueductal grey and area postrema [30]. Brain lesions are present in approximately half of patients at presentation [30, 31], and increase in number with disease progression [30, 32]. We investigated brain MRI findings at onset in all patients in the study. The frequency of abnormal brain MRI findings was 35.9% in NMOSD patients without CTD and 50% in those with CTD; these rates were not significantly different. Nearly all of the supratentorial lesions were non-specific and asymptomatic. These lesions were dot-like or patchy, < 3 cm in diameter, and located in the cerebral deep white matter, as previously described [33]. Infratentorial lesions were more common and specific than supratentorial lesions. In our study, patients had a higher frequency of medulla oblongata lesions than pons or midbrain lesions. This result was consistent with previous reports [28, 34–37]. Although the medulla was the most common brainstem lesion location in NMO, only a few patients showed MRI lesions in the area postrema at onset in our study, which was consistent with the findings of our previous study [10], and all but one of the patients were free of apparent hypothalamic lesions at onset. ‘Classic-’ or ‘typical NMO lesions’ located at the hypothalamus and area postrema are highly specific to the diagnosis of NMOSD; however, these lesions are seen in only a minority of patients, especially at onset [30, 38, 39].

This study had certain limitations. First, as this study is retrospective, bias is inevitable. Second, all the patients came from a single centre, and were insufficient in number. In the future, we will recruit more NMOSD patients with and without CTD and test the titer of AQP4-IgG, which may lead to a deeper understanding of the significance of CTD in NMOSD.

## Conclusions

In conclusion, NMOSD patients with and without CTD were similar in most of the demographic, clinical and laboratory features that we examined. NMOSD patients with CTD have more frequent of T1 hypointensity and T2 BSLs, longer spinal cord lesions on MRI and a lower frequency of short TM than those without CTD.

## Abbreviations

ACA: Anti-neutrophil cytoplasmic antibodies
AHA: Anti-histone antibody
AM: Acute myelitis
ANAs: Antinuclear autoantibodies
ANCAs: Anti-neutrophil cytoplasmic antibodies
anti-dsDNA: Anti-double stranded DNA antibodies
anti-Jo1: Anti-Jo-1 antibodies
anti-nRNP: Antinuclear ribonucleoprotein
anti-Scl70: Anti-topoisomerase I antibodies
anti-Sm: Anti-Sm antibodies
anti-SSA: Anti-SSA/Ro antibodies
anti-SSB: Anti-SSB/La antibodies
AnuA: Anti-nucleosome antibody
AQP4-IgG: Immunoglobulin G autoantibodies against aquaporin-4
ARPA: Anti-ribonucleoprotein antibodies
ASO: Anti-streptolysin
BBB: Blood-brain barrier
BSLs: Bright spotty lesions
C: Complement
CBA: Cell-based assay
CI: Chloride
CNS: Central nervous system
CRP: C-reactive protein
CSF: Cerebrospinal fluid
CTD: Connective tissue disorders
EDSS: Kurtzke Expanded Disability Status Scale
ENAs: Extractable nuclear antigen autoantibodies
FLAIR: Fluid-attenuated inversion recovery
Glu: Glucose
GPI: Glucose-6 phosphate isomerase
IgG: Immunoglobulin G
IVIG: Intravenous immunoglobulin G
IVMP: Intravenous methylprednisolone
LETM: Longitudinally extensive transverse myelitis
MOG-IgG: Myelin oligodendrocyte glycoprotein immunoglobulin G
MRI: Magnetic resonance imaging
NMO: Neuromyelitis optica
NMOSD: Neuromyelitis optica spectrum disorders
NP: Neuropathic pain
OCB: Oligoclonal banding
ON: Optic neuritis
RA: Rheumatoid arthritis
RF: Rheumatoid factor
SD: Standard deviation
SLE: Systemic lupus erythematosus
SPSS: Statistical Package for the Social Sciences
SS: Sjögren syndrome
STIR: Sagittal short tau inversion recovery
T1WI: T1-weighted imaging
T2WI: T2-weighted imaging
TM: Transverse myelitis
UCTD: Undifferentiated connective tissue disorders
VB: Vertebral segments
WBC: White blood cell
